# Appearance and Maturation of T-Cell Subsets During Rat Thymus Ontogeny

**DOI:** 10.1155/1998/24239

**Published:** 1998

**Authors:** A. Vicente, A. Varas, R.S Acedón, E. Jiminez, J. J. Mulqoz, A. G. Zapata

**Affiliations:** Department of Cell Biology Faculty of Biology Complutense University Madrid 28040 Spain

**Keywords:** Ontogeny, rat thymus, T-cell development

## Abstract

In previous papers, we have described the ontogenetical development of thymic stromal-cell components (epithelium, macrophages, dendritic cells) of Wistar rats. Here, we correlate those results with the maturation of rat T-cell precursors along the fetal and postnatal life. First T-cell precursors, which colonize the thymus anlage around days 13-14 of gestation, largely express CD45, CD43, CD53, and Thy 1 cell markers, and in a lesser proportion the OX22 antigen. Rat CD3^-^CD4^-^CD8^-^ thymocytes present in the earliest stages of gestation could be subdivided in three major cell subpopulations according to the CD44 and CD25 expression: CD44^-/+^CD25^-^ → CD44^+^CD25^+^ → CD44^+^CD25^-^ On fetal days 17-18, a certain proportion of CD4^-^CD8^-^cells weakly,express the TcR*β* chain, in correlation with the appearance of the first immature CD4^-^CD8^+^ thymocytes. This cell subpopulation, in progress to the CD4^+^CD8^+^ stage, upregulates CD8*α* before the CD8*β* chain, expresses the CD53 antigen, and exhibits a high proliferative rate. First mature thymocytes arising from the DP (CD4^+^CD8^+^) cells appear on fetal days 20-21. Then, the CD4^+^:CD8^+^ cell ratio is ≤1 changing to adult values (2-3) just after birth. Also, the percentage of V*β*TcR repertoire covered in adult thymus is reached during the postnatal period, being lower during the fetal life. Finally, in correlation with the beginning of thymocyte emigration to the periphery a new wave of T-cell maturation apparently occurs in the perinatal rat thymus.


*Developmental Immunology*, 1998, Vol. 5, pp. 319–331
Reprints available directly from the publisher
Photocopying permitted by license only

© 1998 OPA (Overseas Publishers Association)
N.V. Published by license under the
Harwood Academic Publishers imprint,
part of the Gordon and Breach Publishing Group
Printed in Malaysia

### Appearance and Maturation of T-Cell Subsets During Rat Thymus Ontogeny

A. VICENTE, A. VARAS, R.S ACEDÓN, E. JIMÉNEZ, J. J. MUÑOZ and A. G. ZAPATA,*

[a]*Department of Cell Biology, Faculty of Biology, Complutense University, 28040 Madrid, Spain*

*(Received 22 August 1997; In final form 9 February 1998)*

In previous papers, we have described the ontogenetical development of thymic stromal-cell components (epithelium, macrophages, dendritic cells) of Wistar rats. Here, we correlate those results with the maturation of rat T-cell precursors along the fetal and postnatal life. First T-cell precursors, which colonize the thymus anlage around days 13-14 of gestation, largely express CD45, CD43, CD53, and Thy 1 cell markers, and in a lesser proportion the OX22 antigen. Rat $CD3^-CD4^-CD8^-$ thymocytes present in the earliest stages of gestation could be subdivided in three major cell subpopulations according to the CD44 and CD25 expression: $CD44^{-/+}CD25^-$ → $CD44^+CD25^+$ → $CD44^+CD25^-$. On fetal days 17-18, a certain proportion of $CD4^-CD8^-$ cells weakly express the TcR$\beta$ chain, in correlation with the appearance of the first immature $CD4^-CD8^+$ thymocytes. This cell subpopulation, in progress to the $CD4^+CD8^+$ stage, upregulates CD8$\alpha$ before the CD8$\beta$ chain, expresses the CD53 antigen, and exhibits a high proliferative rate. First mature thymocytes arising from the DP ($CD4^+CD8^+$) cells appear on fetal days 20-21. Then, the $CD4^+$:$CD8^+$ cell ratio is $\leq 1$ changing to adult values (2-3) just after birth. Also, the percentage of V$\beta$TcR repertoire covered in adult thymus is reached during the postnatal period, being lower during the fetal life. Finally, in correlation with the beginning of thymocyte emigration to the periphery a new wave of T-cell maturation apparently occurs in the perinatal rat thymus.

*Keywords:* Ontogeny, rat thymus, T-cell development

*Classification Categories* Abbreviations used:
DN, $CD4^-CD8^-$DP, $CD4^+CD8^+$SP, $CD4^-CD8^+$/$CD4^+CD8^-$F, fetalP, postnatalAD, adult

### INTRODUCTION

The development of the thymus gland is governed by mutual influences between the cell components of thymic stroma and lymphoid-cell progenitors that colonize the organ early during ontogeny. The rat thymic primordium is colonized around fetal day 14 by cell progenitors, the phenotype of which has not been clearly established although the expression on them of different cell markers has been reported

*Corresponding author. Tel.: 34-91-394 4979. Fax: 34-91-394 4981. E-mail: Zapata@eucmax.sim.ucm.es

(Ritter et al., 1978; Paterson and Williams, 1987; Kampinga and Aspinall, 1990; Crook and Hunt, 1996). Moreover, the existence, as reported in mice (Godfrey et al., 1993), of cell subpopulations defined by the expression of CD44 and CD25 in the DN-cell compartment of rats is controversial. CD44, a molecule involved in the homing of cell precursors to mouse thymus has not been described in the rat thymic progenitors and the CD25 expression reported early in ontogeny throughout thymic parenchyma (Habu et al., 1985; Brocke et al., 1987) and, after birth, in the thymic medulla (Habu et al., 1985; Brocke et al., 1987; Kampinga and Aspinall, 1990) have not been associated with DN thymocytes, although IL-2-induced proliferation of rat DN cells has recently been reported (Gotlieb et al., 1993).

The transition from DN-cell compartment to DP cell in Wistar rats is marked by the expression of the CD8 molecule, which allows an intermediate highly proliferative $CD8^{+}CD4^{-}$ population (Paterson and Williams, 1987), which in adult rats seems to weakly express the TcR$\alpha\beta$ (Hünig et al., 1989a, 1989b). On the other hand, the appearance of mature SP ($CD4^{-}CD8^{+}$ and $CD4^{+}CD8^{-}$) thymocytes around birth involves changes in the expression of some cell markers, such as CD45R (Kampinga and Aspinall, 1990) and Thy 1 (Hosseinzadeh and Goldschneider, 1993), which could be related to the emigration of first T lymphocytes to the periphery. Two other relevant events occur in the rat thymus during the perinatal period. As previously reported in mice, there is an important increase of thymic cellularity that could be associated with *in situ* increased proliferation (Ceredig, 1990; Lawetzky et al., 1991) and/or with the arrival at the organ of a second wave of T-cell precursors (Penit and Vasseur, 1989). In addition, the CD4/CD8 cell ratio gradually changes during perinatal life to the adult condition. In mice, this phenomenon has been correlated with a distinct capability of the embryonic and adult T-cell precursors to differentiate (Adkins, 1991) and with the existence of different mechanisms of thymic selection in each period due to the presence or absence of TdT activity in adult or fetal precursor cells, respectively (Shortman and Wu, 1996).

Despite this available evidence, a systematic analysis of the ontogenetical maturation of both the lymphoid and nonlymphoid cell populations of rat thymus is, to our knowledge, lacking, although Kampinga and Aspinall (1990) carried out an immunohistochemical study on rat thymus ontogeny and Hünig et al. (1989a) analyzed by flow cytometry the expression of some rat T-cell markers, including CD2, TcR$\alpha\beta$, and CD25 (IL-2R$\alpha$ chain) in the last stages of fetal life. Previously, we studied the ontogeny of rat thymic stromal-cell components, including epithelial cells, dendritic cells, and macrophages (Vicente et al., 1994, 1995, 1996). In the present study, we combine both immunohistochemistry and flow cytometry to analyze the appearance and differentiation of distinct rat T-cell subsets and the influence of the thymic nonlymphoid cell microenvironments on these processes.

### RESULTS

### Evolution of Thymic-Cell Numbers During Ontogeny

Between days 15-21 of embryonic life, the cell number exponentially increased in the thymus of Wistar rats. At birth, it remained constant, and from day 2 of postnatal life onwards, the rat thymus gland underwent a new exponential growth to reach the adult condition (Table I). On the other hand, evolution of the thymic-cell numbers correlated well with the frequency of cycling cells in each studied stage. Between fetal days 16-19, there was a high percentage of cells in S + G2 + M phases, decreasing rapidly at day 21. Around birth, the percentage of cycling cells increased transiently, decreasing again to adult values by the second week of postnatal life (Table I).

### The Earliest Thymocytes

As previously reported (Vicente et al., 1996), the first T-cell precursors reached the rat thymus anlage at days 13-14 of gestation. These progenitor cells expressed CD45, CD53, and CD43 antigens (Figure 1a). On days 15-16, flow cytometry analysis showed

TABLE I Evolution of Thymus Size and Cell Proliferation During Embryonic and Postnatal Wistar Rat Development

| Days | Thymic cellularity ($\times 10^{-6}$) | % of cells in S + G2 + M phases |
|---|---|---|
| **15F** | 0.006 ± 5 × $10^{-4}$ | 32 ± 3.0 |
| **16F** | 0.033 ± 0.002 | 33 ± 2.4 |
| **17F** | 0.21 ± 0.01 | 44 ± 4.4 |
| **18F** | 1.29 ± 0.1 | 52 ± 3.7 |
| **19F** | 3.51 ± 0.3 | 44 ± 2.4 |
| **20F** | 6.16 ± 0.9 | 21 ± 3.1 |
| **21F** | 7.47 ± 1.1 | 12 ± 2.1 |
| **22F** | 7.44 ± 0.9 | 22 ± 3.1 |
| **1P** | 7.55 ± 1.3 | 23 ± 3.3 |
| **2P** | 8.25 ± 1.2 | 21 ± 4.5 |
| **3P** | 13.2 ± 2.5 | 19 ± 3.9 |
| **7P** | 60 ± 10 | 13 ± 2.1 |
| **AD** | 850 ± 47 | 10 ± 2.3 |

*Note*: Mean of six independent determinations ± SEM. Five to ten pooled thymuses before birth.
Abbreviations: F = fetal; P = postnatal; AD = adult.

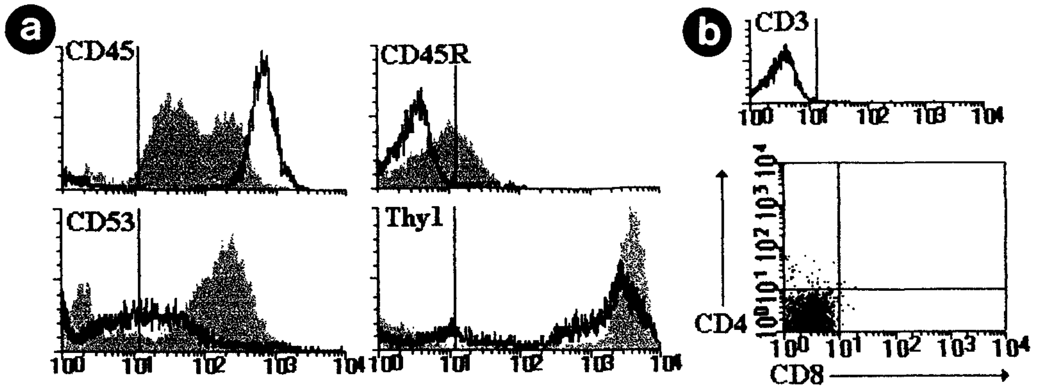

FIGURE 1 Phenotype of the earliest thymocytes occurring in the rat thymic primordium. (**a**) Expression of different antigens (CD45, CD45R, CD53, and Thy 1) on 15 (solid profile) and 17- (line profile) day-old fetal rat thymocytes. (**b**) Thymocytes from 15-day-old fetal rats were triple-labeled for CD4 versus CD8 and CD3 cell markers.

that most thymocytes corresponded to $CD4^-CD8^-CD3^-$, triple-negative (TN) cells (Figure 1b), strongly expressing Thy 1 antigen (60-65%) (Figure 1a). In addition, the mAb OX22, which identifies high-molecular-weight isoforms of CD45, reacted with a significant population of thymocytes (20-25%) on fetal days 15-16 (Figure 1a). Because neither B cells nor mature thymocytes, known to express the OX22 epitope, were noticed in the rat thymus until fetal day 20 (data not shown), these early $OX22^+$ cells probably correspond to T-cell precursors.

In the following developmental stages, the percentage of both $CD45^+$ and $CD43^+$ cells remained constant, although the level of expression of both antigens varied (Figure 1a). On the contrary, the proportion of $CD53^+$ and $OX22^+$ cells decreased, whereas the frequency of Thy $1^+$ cells remained unchanged on fetal days 17-18, although the proportion of the Thy $1^{lo}$ cells increased (Figure 1a). From day 19 onwards, the pattern of Thy 1 expression was similar to that found in adult thymus, with most cells being Thy $1^{hi}$.

### Interleukin-2 Receptor $\alpha$ Chain (IL-2R$\alpha$) Expression During Thymus Ontogeny

The number of IL-2R$\alpha$ chain (CD25)-positive cells reached maximal values in 16-day-old fetal thymus (Figure 2a), although the expression of this cell marker was already cytometrical and immunohistologically detectable on day 15 of embryonic life (Figures 2b and 2c). The proportion of IL-2R-positive cells gradually declined until day 20, peaking again in the perinatal period (Figure 2a).

On the other hand, the expression of CD25 and CD44 antigens allowed to define three major cell subpopulations in the 15- to 17-day-old fetal thymus: $CD44^{-/+}CD25^{-}$, $CD44^{+}CD25^{+}$, $CD44^{+}CD25^{-}$ (Figure 2c). Furthermore, the CD25 expression on $CD44^{+}CD25^{+}$ cells showed an important heterogeneity. The $CD25^{hi}$ cell subset predominating on days 15-16 rapidly diminished in parallel with the acquisition of the T-cell maturation associated molecules (Figure 2c). Moreover, 30-40% of total $CD25^{+}$ cells expressed neither Thy 1 nor CD2 antigens (Figure 2d).

### Acquisition of Cell Markers Associated with T-Cell Maturation

From the first day studied (day 15), 20-30% of thymocytes expressed CD2 antigen. The numbers of $CD2^{+}$ cells rapidly increased (day 16: 60-70%) to

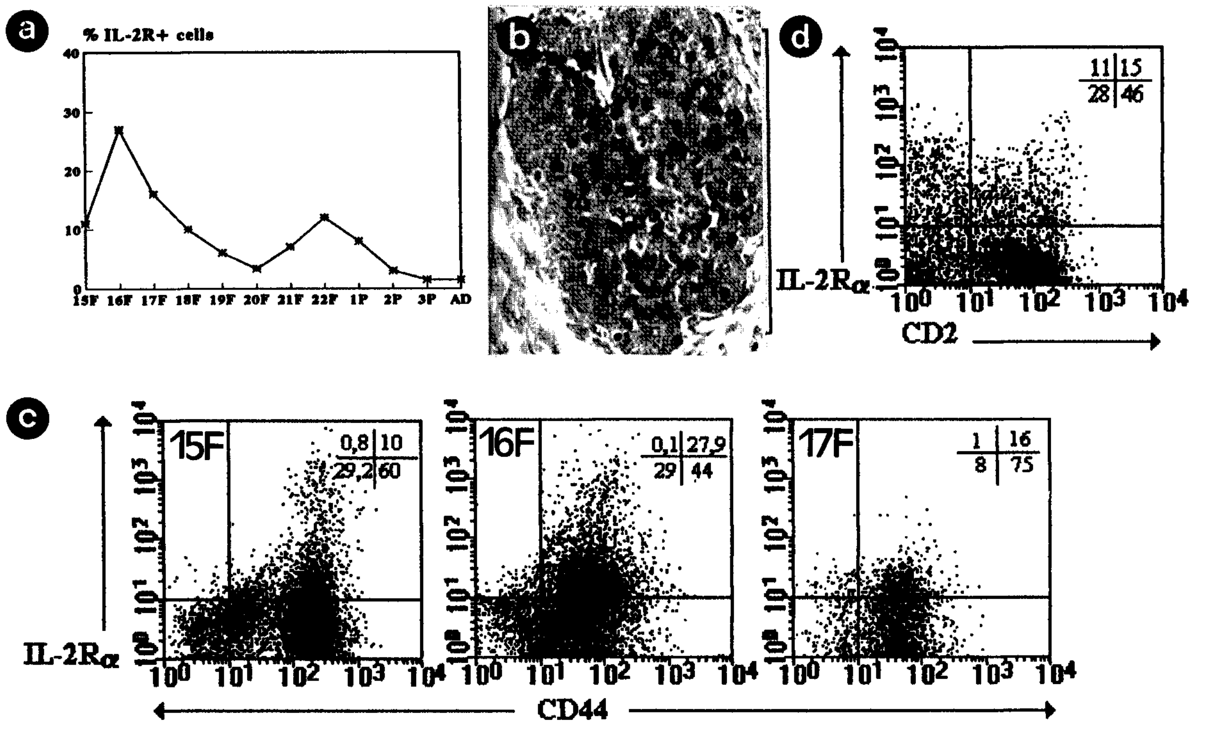

FIGURE 2 IL-2R$\alpha$ (CD25) expression in rat early thymocytes. (**a**) Frequencies of $CD25^{+}$ cells in the rat thymus during ontogeny. Two discrete peaks are evident on 15-16 embryonic days and around birth. (**b**) CD25-positive thymocytes scattered throughout the thymic parenchyma of a 15-day-old embryonic rat. Magnification: 50 X. (**c**) The analysis of cell-surface expression of CD44 versus CD25 cell markers on earliest thymocytes suggests the following development sequence of rat fetal DN thymocytes: $CD44^{-/+}CD25^{-} \rightarrow CD44^{+}CD25^{+} \rightarrow CD44^{+}CD25^{-}$. The percentages of each subpopulation are listed at the top right corner of the dot plot. These profiles are representative of over five independent experiments. (**d**) CD2 versus CD25 expression on 16-day-old fetal thymocytes.

reach adult values on days 17-18 (90-95%). On the contrary, the first $CD5^+$ cells did not appear until day 18 (70-75%), increasing in the following days (80-85%), although at no stage studied was the number of $CD5^+$ cells higher than that of $CD2^+$ thymocytes.

Although the flow cytometry analysis did not reveal $CD8^+$ thymocytes in the 16-day-old fetal thymus (Figure 3a), positive cells were able to be detected throughout the organ by immunohistochemistry (data not shown), demonstrating the cytoplasmic expression of this antigen. One day later, about 40-45% of total thymocytes already expressed a surface CD8 $\alpha$ chain, whereas a $\beta$ chain was only detected in half of these cells (Figure 3b). In the following stages, most $CD8^+$ cells (98%) expressed both $\alpha$ and $\beta$ chains (data not shown). In addition, on fetal days 17-18, most immature $CD4^-CD8^+$ cells expressed the CD53 antigen (Figure 3c), and showed a high proliferative rate that accounted for the increased numbers of total cycling cells found in these developmental stages (Figure 3d). This immature $CD4^-CD8^+$ cell population preceded the appearance of $CD4^+CD8^+$ double-positive (DP) and mature $CD4^-CD8^+/CD4^+CD8^-$ single-positive (SP) cells, which occurred on fetal days 18-19 and 20-21, respectively (Figure 3a).

### Expression of TcR$\alpha\beta$ on Thymocytes

The first TcR$\alpha\beta$-positive cells were immunohistologically identified scattered throughout the thymus on day 17, 1 day before (day 18) that TcR$\alpha\beta$ surface expression was detected by flow cytometry (Table II).

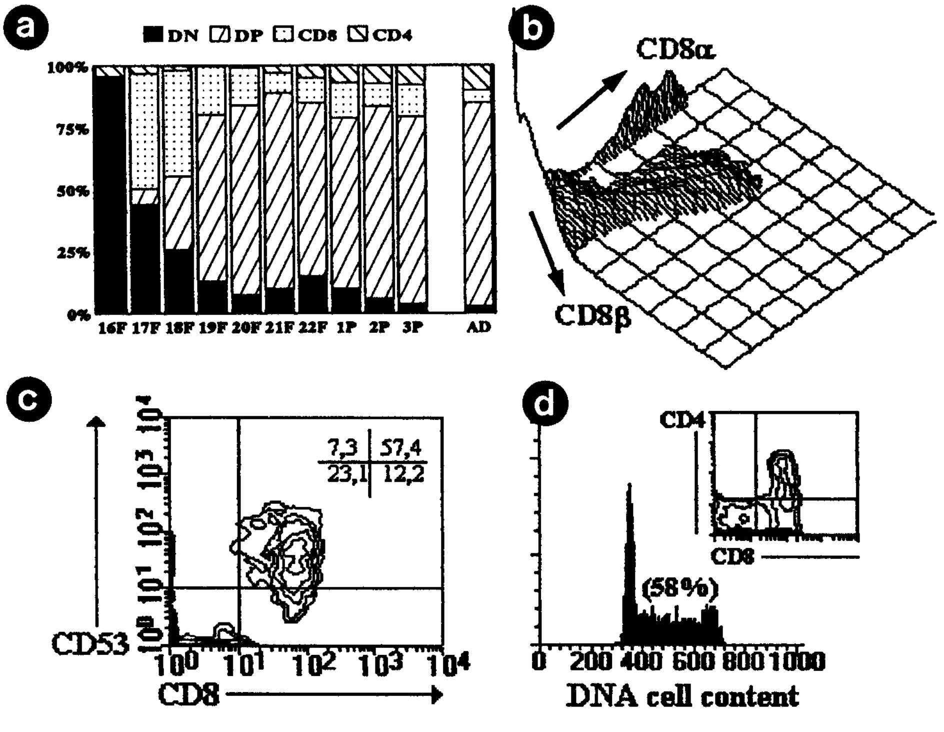

FIGURE 3 (a) Evolution of rat thymocyte subpopulations defined according to CD4- and CD8-cell-marker expression. Thymocyte suspensions were prepared from embryonic, newborn, and adult thymuses, and analyzed for CD4 versus CD8 expression. Values represent the mean of over five independent tests. (b) CD8$\alpha$ and $\beta$-chain surface expression on the total thymocytes from 17-day-old embryonic rats. Note the existence at this stage of a numerically important $CD8\alpha^+\beta^-$ cell subpopulation. (c) Expression of CD8 versus CD53 on thymocytes from 18-day-old embryonic rats. Percentages of the different subpopulations are included in the right top corner. (d) Three-color flow cytometry analysis of the total thymocytes from 18-day-old embryonic rats. CD4 versus CD8 expression is represented in the dot plot. Percentage of the $CD4^-CD8^+$ gated cells in S and $G_2$ + M phases of the cell cycle after propidium iodide staining.

TABLE II Expression of TcR$\alpha\beta$ During Rat Thymus Ontogeny

| | 18F | 19F | 20F | 21F | 22F | 1P | 2P | 7P | AD |
|---|---|---|---|---|---|---|---|---|---|
| **TcR$^{lo}$** | 46 ± 5 | 55 ± 3 | 53 ± 4 | 61 ± 4 | 50 ± 4 | 50 ± 5 | 53 ± 4 | 63 ± 2 | 62 ± 4 |
| **TcR$^{hi}$** | - | 2 ± 1 | 7 ± 1 | 14 ± 3 | 17 ± 3 | 16 ± 2 | 14 ± 2 | 7 ± 1 | 8 ± 2 |

*Note*: Percentages of TcR$\alpha\beta^{lo}$- and TcR$\alpha\beta^{hi}$-cell subpopulations in fetal, postnatal, and adult thymus of Wistar rats. Values represent the mean ± SEM of five independent tests.
Abbreviations: F = fetal; P = postnatal; AD = adult.

Gradually, the percentage of TcR$\alpha\beta$/CD3$^+$ cells increased with the first CD3/TcR$\alpha\beta^{hi}$ cells appearing on days 19-20 (Table II). In the perinatal period, the proportion of TcR$\alpha\beta^{hi}$-expressing cells suddenly increased to further reach adult values by the beginning of the second week of postnatal life (Table II). This increase of mature thymocytes correlated well with a high incidence of dividing cells (25-30%) observed in the TcR$\alpha\beta^{hi}$-cell subset during the perinatal period. On the contrary, only 4-6% TcR$\alpha\beta^{hi}$ cells proliferated in 2-week-old thymus. In addition, mature thymocytes quickly upregulated the expression of other surface antigens, including CD45R, CD43, CD53, CD5, and CD25 (data not shown).

A three-color flow cytometrical analysis was carried out to examine the distribution of TcR$\alpha\beta^{lo}$ and TcR$\alpha\beta^{hi}$ thymocytes among CD4- and CD8-defined subpopulations. In all stages studied, TcR$\alpha\beta^{lo}$ cells largely corresponded to DP (50-60%) and CD4$^-$CD8$^+$ (30-45%) thymocytes. However, on fetal day 18, when the first TcR$\alpha\beta^+$ cells were detected by flow cytometry, around 30-40% CD4$^-$CD8$^-$ (DN) cells expressed low levels of T-cell receptor (Figure 4a). The proportion of DN TcR$\alpha\beta^{lo}$ cells dropped drastically (2-7%) in the following days (Figure 4a). Furthermore, a DN-cell subset expressing intermediate levels of TcR$\alpha\beta$ was identified from the second week of postnatal life, but not during the embryonic period (Figure 4a). On fetal days 19-20, TcR$\alpha\beta^{hi}$ cells consisted of a significant proportion of DP (60-80%) and CD4$^-$CD8$^+$ (20-30%) cells (Figure 4b). Only a few of these cells (12-15%) were, however, CD4$^+$CD8$^-$ on day 20 (Figure 4b). In contrast, during the first week of postnatal life, the major percentage of TcR$\alpha\beta^{hi}$ cells belonged to the CD4$^+$CD8$^-$ cell subset (40-50%), as detected in the adult thymus (Figure 4b). Thus, 20-day-old fetal thymocytes showed a lower CD4:CD8 ratio (~0.43) than that achieved by thymocytes from 1-week-old neonatal rats (~1.5-3).

Further analysis of the TcR$\alpha\beta^{hi}$-cell subset using a limited panel of mAb specific to rat V$\beta$ gene products, showed similar values for the different V$\beta$ TcR$^{hi}$ cell subsets on fetal days 19-20 (Figure 5). In correlation with the increased numbers of total TcR$\alpha\beta^{hi}$-expressing cells that occurred around birth, there was an increase of TcRV$\beta^{hi}$ thymocytes, especially in the V$\beta$8.5- and V$\beta$8.2-cell subsets (Figure 5). Remarkably, in the following week of postnatal life, the proportion of both thymocyte subsets underwent a profound reduction, reaching adult values only on the second and third week, respectively. On the contrary, the V$\beta$10- and V$\beta$16-cell subpopulations remained practically unchanged from birth onwards, slightly increasing from the second week of postnatal life (Figure 5).

### Perinatal Increase of Immature Thymocytes

Along with the high numbers of mature thymocytes occurring in the perinatal period, there was a transitional increase in both the absolute numbers and the proportion of CD4$^-$CD8$^-$ cells (Figure 3a), which, unlike SP thymocytes, was not accompanied by an increased proliferative rate (always around 25-30% until the end of the first week of postnatal life). An increase in immature CD8$^+$ thymocytes, as well as a notable reduction of DP and CD3/TcR$\alpha\beta^{lo}$ cell subsets also occurred in this period (Figure 3a, Table II).

### DISCUSSION

Rat thymus anlage is colonized by cell precursors around days 13-14 of gestation (Vicente et al., 1996). These cells largely expressed CD45, CD43, and CD53 antigens. A similar phenotype has recently been described in cell progenitors from early fetal rat liver (Crook and Hunt, 1996), suggesting that cells reaching the rat thymic rudiment could already express these cell markers. The expression of high-Mr isoform of the leukocyte common antigen (CD45) has been demonstrated in adult human (Deans et al., 1991), mouse (Goff et.al., 1990), and rat (Law et al., 1989) DN cells, and precursor-cell activity largely reside in the CD45RA$^+$ fraction of adult DN thymocytes (Law et al., 1989; Goff et al., 1990). On the contrary, the expression of high-Mr isoforms of CD45 on T-cell precursor during development is unclear. Whereas some authors (Law et al., 1989; Kampinga and Aspinall, 1990) were unable to identify OX22$^+$ cells in the early rat thymic primordium, our flow cytometry analysis demonstrates, as in adult thymus, the presence of a CD45R$^+$ (OX22-reacting cells) thymocyte subset on days 15-17 of gestation, which rapidly decreases in the following days in correspondence to T-cell maturation.

The arrival of cell progenitors into the rat thymic primordium (a homogeneous mass of primitive epithelial cells) results in the differentiation of several ultrastructurally distinct epithelial-cell types, the

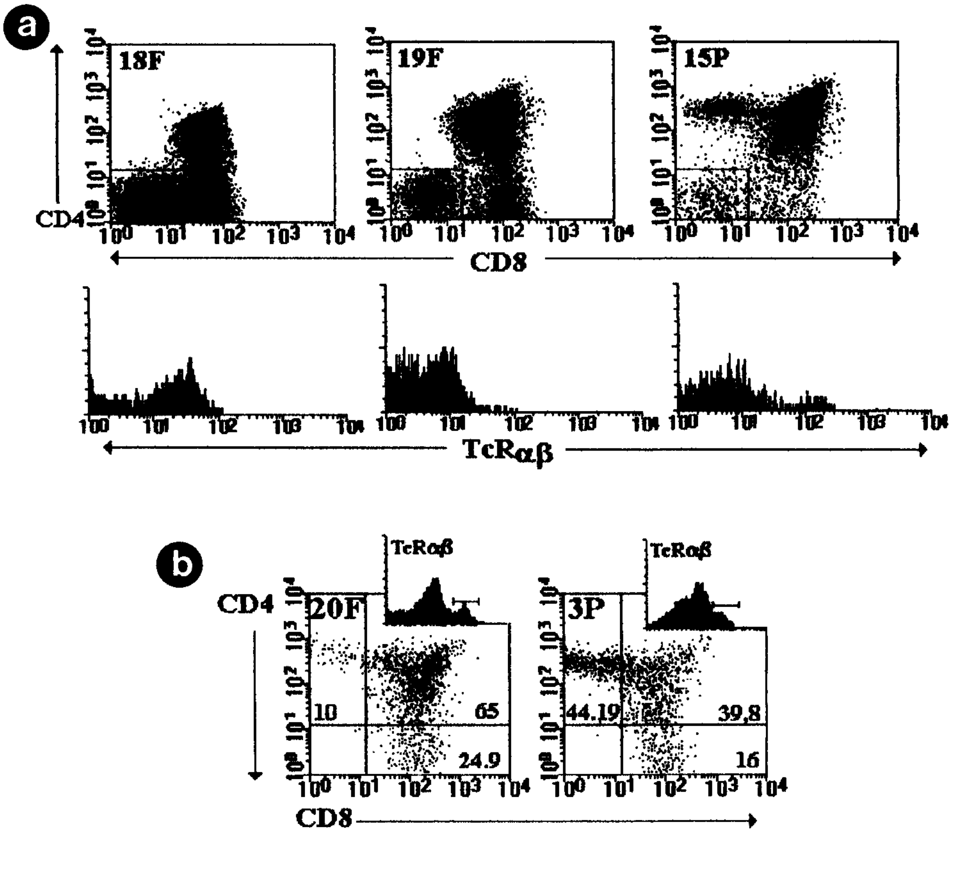

FIGURE 4 (a) TcR$\alpha\beta$ expression on DN rat thymocytes during fetal and postnatal development. DN cells were gated as indicated in the CD4 − CD8 dot plot. (b) Percentage of TcR$\alpha\beta^{hi}$ thymocytes either from 20-day-old embryonic rats or 3-day-old postnatal rats that express CD4 and/or CD8. TcR$\alpha\beta^{hi}$ was defined as indicated in the histogram. Two thousand TcR$\alpha\beta^{hi}$ gated events are shown.

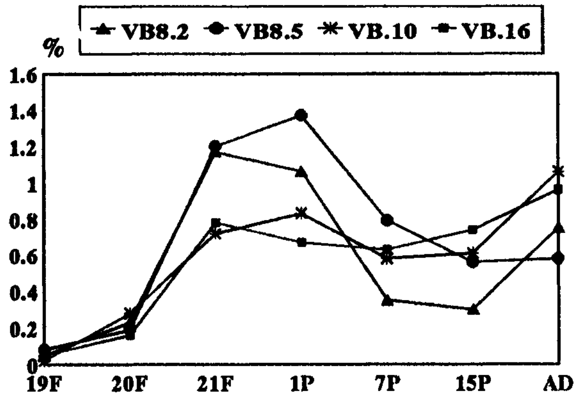

FIGURE 5 Frequency of Vβ$TcR^{hi}$ thymocytes during rat fetal and postnatal development. Values represent the mean of three to five independent determinations.

expression on them of MHC class I and class II molecules, and the acquisition of first specific cortical- and medullary epithelial-cell markers (Vicente et al., 1996). Other authors have emphasized the relevance of cell-progenitor colonization for inducing growth and differentiation of the thymic epithelium (Haynes and Heinly, 1995). On the other hand, the interactions between developing epithelial cells and T-cell precursors presumably induce the proliferation of the latter, the appearance of a pre-TcR, the expression of CD8$\alpha$ and then CD8$\beta$ chain, and, finally, the progression to the DP stage. Meanwhile, surface-cell markers strongly expressed on TN cells, such as CD43, CD53, CD45R, and CD25 are totally or partially lost.

The relevance of IL-2/IL-2R complex for T-cell maturation is a controversial issue. The IL-2R$\alpha$ chain is detected in a high proportion of adult and fetal DN cells from humans, mice, and chickens (Ceredig et al., 1985; Toribio et al., 1989; Fedecka-Brunner et al., 1991; Kondo et al., 1993). However, previous works have failed to demonstrate IL-2R$\alpha$ expression on adult and fetal rat DN thymocytes (Tackacs et al., 1988; Kampinga and Aspinall, 1990). On the contrary, and in agreement with our results, Brocke et al. (1987) immunohistochemically detected $CD25^+$ cells in early embryonic rat thymus. These authors proposed, however, that they could be DP and/or mature SP cells, because the thymic lobes also expressed CD4 and CD8 molecules. In fact, the expression of these antigens at early stages of development is clearly cytoplasmic, as shown by our flow cytometrical and immunohistochemical results, corresponding to late DN cells differentiating to the DP-cell compartment. Our data demonstrate, therefore, that the CD25 expression in rat follows an analogous kinetics and distribution to that described for embryonic mice (Ceredig et al., 1985) and chickens (Fedecka-Brunner et al., 1991), and indirectly suggest certain functional relevance of the IL-2/IL-2R complex during rat thymic ontogeny. In support, the addition of IL-2 or anti-CD25 mAbs to 16-day-old fetal rat thymus organ cultures stimulated or inhibited, respectively, the proliferation and differentiation of immature thymocytes (Varas et al., 1997b).

On the other hand, a significant proportion of early $CD25^+$ thymocytes do not express either Thy 1 or CD2. As suggested by the current results and by those in mouse thymus (Mertsching and Ceredig, 1996), Thy $1^{-/lo}$ $CD2^-$ progenitor cells colonize the thymic primordium and rapidly differentiate into a more mature Thy $1^{hi}$ $CD2^+$ cell population. Thus, this evidence is consistent with the idea of CD25 being expressed on T-cell precursors immediately after their arrival to the thymic primordium. Furthermore, the analysis of CD44 and CD25 expression on 15- to 17-day-old fetal rat thymus defined various $CD4^-CD8^-CD3^-$ triple-negative (TN) cell subpopulations, the sequence of maturation of the first cohort of fetal rat thymocytes being: $CD44^{\mp}CD25^-$ → $CD44^+CD25^+$ → $CD44^+CD25^-$. This developmental sequence has not been, to our knowledge, described in TN cells of adult rats and it is different from that reported in adult mice (Godfrey et al., 1993). This could suggest that fetal thymocytes may follow different kinetics of maturation than adult ones, as previously pointed out in fetal mice (Andjelic et al., 1993).

In mice, previous results have shown that TcR$\beta$ gene rearrangement is necessary for the progression from DN- to DP-cell compartment (Levelt and Eichmann, 1995). In agreement, using R.73 mAb to a constant determinant of the rat TcR$\alpha\beta$ (Hünig et al., 1989b), we found an important proportion of $R.73^+$

DN cells in early developmental stages of Wistar rat thymus, which decreases during ontogeny. According to the TcR$\alpha\beta^{lo}$ expression on this DN-cell subpopulation and since rat TcR$\alpha$ mRNA is only detectable from the onset of the $CD4^+CD8^+$ stage (Park and Hünig, 1995), we propose that the R.73$^+$ DN-cell subpopulation could correspond to immature $CD4^-CD8^-$ cells bearing a TcR$\beta$ chain, as also reported in adult and fetal immature mouse thymocytes (Groettrup et al., 1993; Wilson and MacDonald, 1995). Moreover, the mature DN-cell subset, which expresses intermediate levels of TcR$\alpha\beta$, does not appear in the embryonic thymus, being only detectable after birth (our own results, Fowlkes et al., 1987).

As in human thymus (Haynes and Heinly, 1995), CD8$\alpha$ chain is expressed earlier than CD8$\beta$ chain in fetal rat thymus. In fact, rat $CD4^-CD8^-$-cell subpopulations progress to the $CD4^+CD8^+$ stage through an intermediate $CD4^-CD8^+$ cell that appears for the first time on day 17 of gestation. This cell subpopulation represents less than 2% of total thymocytes in adult rats (Paterson and Williams, 1987), whereas in fetal days 17-18 it, represents 40-50% of total thymic cells. Furthermore, in adult rats, immature $CD4^-CD8^+$ thymocytes can be distinguished from mature $CD8^+$ cells by the lack of OX44 expression (Paterson and Williams, 1987). In contrast, in the fetal thymus, the loss of OX44 expression does not occur until the DP stage. The physiological relevance of this phenotypical difference is currently unknown. In addition, $CD4^-CD8^+$ cells show a high proliferative rate in any condition analyzed (our own results; Paterson and Williams, 1987; Penit and Vasseur, 1989) suggesting that this intermediate-cell subset significantly contributes to the exponential growth of early rat thymus.

Despite their high proliferative rate, $CD4^-CD8^+$ immature cells have an extremely limited lifespan, quickly progressing to DP cells, which, thus, on fetal days 19-20 constitute the major thymic cell subpopulation. Their appearance involves the fully maturation of thymic epithelial cells, the histological differentiation of a thymic cortex and the increase of both expression and number of MHC class I and class II positive cells (days 18-19) (Vicente et al., 1996). The upregulation of TcR$\alpha\beta$/CD3 complex in the DP cells and the strong expression of MHC molecules on both epithelial cells and dendritic cells (Vicente et al., 1994, 1996) allow the intrathymic thymocyte selection. Nonselected apoptotic cells are eliminated by thymic macrophages, which mature in the last days of fetal life (Vicente et al., 1995).

Positively selected $CD4^+CD8^+$ cells produce the first mature SP thymocytes that accumulate in the central area of the organ defining the thymic medulla around day 20 of gestation. The analysis of rat SP thymocytes in both perinatal and adult conditions showed important differences. In agreement with previous reports (Ceredig, 1990), we detected a high incidence of perinatal TcR$\alpha\beta^{hi}$ thymocytes in S + G2 + M phases, whereas adult mature thymocytes showed a low cycling activity. This increased intrathymic proliferation of mature thymocytes could play an important role in providing a sufficiently large number of T lymphocytes leaving the thymus to colonize the peripheral lymphoid organs during the perinatal period. Accordingly, in the neonatal mouse thymus, the proportion of thymic emigrants is higher than in the adult thymus (Weissman, 1967). Moreover, as demonstrated in mice (Modigliani et al., 1994), the low proliferative rate of peripheral T cells in the neonatal period implies that in those stages, the peripheral expansion of T lymphocytes is basically a consequence of the increased thymic-cell emigration. The nature of the signal(s) promoting the increased thymocyte proliferation in perinatal thymus is yet unknown. Ceredig and Waltzinger (1990) proposed the involvement of some cytokines in the process. In agreement, we have recently demonstrated that in rat fetal thymus organ cultures (FTOC) supplemented with either IL-2 or IL-7 for 24 hr at different times of culture, the highest responses of mature SP thymocytes occur in those days of culture equivalent to the *in vivo* perinatal stage (Varas et al., 1997a, 1997b).

In correlation with the beginning of thymocyte emigration to the periphery, a new wave of T-cell maturation occurs in the perinatal rat thymus, a fact previously described in mice (Penit and Vasseur, 1989). Since the percentage of DNA synthetizing DN

cells remained constant throughout ontogeny (our own results, Penit and Vasseur, 1989), and the proportion of early immature thymocytes increases around birth, a new wave of precursor cells could be colonizing the rat thymus during the perinatal period. Remarkably, at that time, we demonstrated enlarged perivascular spaces throughout the thymic parenchyma, implying an increased thymic vascular permeability (Vicente et al., 1995), which could favor the emigration of mature thymocytes as well as the arrival of new cell precursors. Periodic renewal of the intrathymic progenitor-cell subpopulation has been previously described during embryonic life in both mice and birds (Jotereau et al., 1987). Most probably, a dynamic equilibrium between homing-cell precursors and emerging mature cells occurs (Zuñiga-Pflücker and Lenardo, 1996).

Other events that occur around birth in rat thymus could be related to the proliferative activity of perinatal thymocytes. Our results demonstrate that the percentage of repertoire covered in adult thymus (24-28%) was reached in the postnatal period, whereas only 17-19% of repertoire was covered in 20-day-old embryos. Remarkably, there is a preferential expansion of specific TcRV$\beta$-cell subpopulations during the perinatal period. Thus, between days 20-21 of gestation, the numbers of total TcR$\alpha\beta^{hi}$ thymocytes increased 3.6 times, similar to V$\beta$10TcR$^{hi}$ cells (3.3 times), and V$\beta$8.5TcR$^{hi}$ thymocytes showed the greatest expansion (7.5 times). V$\beta$8.2- and V$\beta$16TcR$^{hi}$-cell subsets increased in a lesser extent (6.1 and 5.8 times, respectively). Presumably, the distinct expansion of these mature T-cell subsets is correlated with differential responses to cytokines, for which they express specific receptors, as previously pointed out in humans (He and Kabelitz, 1993). This selective response has been recently confirmed by us in either IL-2- or IL-7-treated rat FTOC (Varas et al., 1997a, 1997b).

On the other hand, as previously reported (Adkins, 1991), during fetal development, fewer mature CD4$^+$ than CD8$^+$ T cells are produced. In contrast, in both postnatal and adult thymus, the frequency of CD4$^+$ cells is higher. Adkins and Hamilton (1994) explained that the proportions of CD4$^+$ cells would be regulated by a combination of the developmental ages of the T-cell precursors and the thymic stromal environment. Accordingly, during ontogeny, a unique communication system could operate between fetal thymic stroma and fetal progenitors, which would generate the decreased CD4/CD8 ratio. Recently, Shortman and Wu (1996) have speculated that differential production of CD4$^+$ and CD8$^+$ T cells could reflect the presence or absence of TdT in adult or fetal precursor cells, respectively. Accordingly, adult TdT$^+$ progenitors could lead to more MHC class II-restricted receptors appropiate for CD4$^+$ lymphocytes. We propose, however, that initially, the transition from fetal to adult CD4/CD8 ratios could reflect a higher proliferative rate of CD4$^+$ thymocyte subpopulation, compared to mature CD8$^+$ cells, since the typically decreased CD4/CD8 ratio at days 20-21 of gestation changes as soon as by 1 day of postnatal life.

### MATERIALS AND METHODS

### Animals

Wistar rat thymus glands were sampled from day 15 of fetal life to 2 weeks after birth. Adult animals were also included in the study.

### Antibodies

Reagents included either unconjugated, FITC- or PE-conjugated anti-CD45 (OX-1), anti-CD45R (OX-22), anti-CD43 (W3/13), anti-Thyl (OX-7), anti-CD53 (OX44), anti-CD44 (OX49), anti-CD25 (OX-39), anti-CD2 (OX34), anti-CD5 (OX-19), anti-CD4 (OX-38), anti-CD8$\alpha$ (OX-8), anti-CD8$\beta$ (3.41), anti-CD3 (G4.18), and anti-TcR$\alpha\beta$ (R.73) (all from Pharmingen, San Diego, California). A panel of mAbs to rat TcR V$\beta$8.2 (R.78), V$\beta$8.5 (B73), V$\beta$10 (G101), and V$\beta$16 (His 42) gene products was kindly provided by Dr. T. Hünig (Würzburg University, Würzburg, Germany) and Dr. J. Kampinga (Groningen University, Groningen, The Netherlands).

### Cell Suspensions

Thymi were aseptically removed from adults, neonates and 15- to 22-day-old fetuses, these latter using a stereoscopic microscope. Single-cell suspensions of thymocytes were prepared by pressing disrupted thymic lobes through a steel sieve and maintained on ice in PBS with 1% FCS before use. Cell viability was assessed by using Tripan-blue and cell number determined in duplicate using a hemocytometer.

### Cell-Surface Staining

Phenotypic analysis of thymocytes recovered from different stages was performed as follows: $1 \times 10^5$ cells were incubated with saturating amounts of antibodies for 20 min at 4°C. Two-color immunofluorescence labeling was done by incubating with a mixture of FITC- and PE-conjugated mAbs. For three-color analysis, cells were sequentially exposed to (i) unconjugated anti-TcR$\alpha\beta$ or anti-TcRV$\beta$ monoclonal antibodies, (ii) tricolor-conjugated $F(ab')_2$ fragment of goat anti-mouse IgG, (iii) normal mouse serum (1:100), and (iv) anti-CD8-FITC and anti-CD4-PE mAbs. Background fluorescence was determined using an isotype-matched control mAb. Stained cells were analyzed using a FACScan (Becton Dickinson, San José, California). Dead cells were excluded from data acquisition on the basis of forward/side scatter and in two-color stainings, by staining with propidium iodide. The data were analyzed using PC-LYSIS software (Becton Dickinson).

### Cell-Cycle Analysis

Cell-cycle analysis was carried out by using $1 \times 10^6$ cells fixed for 7 min in 70% ethanol at −20°C, washed in Tris-HCl buffer to pH 6.0, incubated with a RNAse dilution (1mg/ml Tris-HCl) for 30 min at 37°C, washed in Tris-HCl buffer and resuspended in a solution of 0.05 mg/ml propidium iodide in PBS. In some cases, in order to assess the proliferative rate of various thymocyte subpopulations throughout ontogeny, cells were firstly stained with FITC-TcR$\alpha\beta$ or FITC-CD8 and PE-CD4 according to the previously described protocol. Analysis was carried out in a FACScan, using Cell Fit software (Becton Dickinson).

### Immunohistochemistry

Thymic lobes were snap frozen in liquid nitrogen and stored at −80°C until use. Five-micrometer-thick cryosections were fixed for 10 min in acetone and then incubated with different primary mAbs. Endogenous peroxidase was blocked with 1% $H_2O_2$ in methanol for 15 min. After washing in PBS, the histological sections were incubated with 1:40 solution of peroxidase-conjugated rabbit anti-mouse Ig in PBS (Dakopatts, Glostrup, Denmark) supplemented with 1:100 normal rat serum. After washing, the peroxidase reaction was developed with 0.05% 3,3′ diaminobenzidine (Sigma, St. Louis, MO) in PBS with 0.1% $H_2O_2$ for 10 min. Sections were counterstained with methylene blue. Preparations incubated without primary antibodies were used as negative controls.

### *Acknowledgments*

The authors wish to thank the following people for providing monoclonal antibodies: Dr. T. Hünig (Würzburg University, Germany) and Dr. J. Kampinga (Groningen University, The Netherlands). This work was supported in part by the Spanish Ministry of Education and Culture, grant numbers CICYT PB 91-0374 and PB94-0332. R. Sacedón, E. Jiménez and J. J. Muñóz are recipients of fellowships from the Spanish Ministry of Education and Culture.

The flow cytometric analysis was performed on a FACScan flow cytometer from the Research Center, Faculty of Biology, Compluteuse University.

### *References*

Adkins B. (1991). Developmental regulation of the intrathymic T cell precursor population. *J. Immunol.* **146**, 1387-1393.

Adkins B., and Hamilton K. (1994). Developmental ages of the thymic epithelium and of the T cell precursors together determine the proportions of peripheral CD4+ cells. *J. Immunol.* **153**, 5359-5365.

Andjelic S., Jain N., and Nicolic-Zugic J. (1993). Ontogeny of fetal $CD8^{lo}CD4^{lo}$ thymocytes: Expression of CD44, CD25 and early expression of TcR$\alpha$ mRNA. *Eur. J. Immunol.* **23**, 2109-2115.

Brocke S., Takacs L., Gerdes J., Osawa H., and Diamantstein T. (1987). The ontogeny of the IL-2R expression and of the IL-2 responsiveness in the rat thymus. *Immunobiol.* **174**, 266-273.

Ceredig R. (1990). Intrathymic proliferation of perinatal mouse $\alpha\beta$ and $\gamma\delta$ T cell receptor-expressing mature T cells. *Int. Immunol.* 2, 869-867.

Ceredig R., Lowenthal J.W., Nabholz M., and MacDonald H.R. (1985). Expression of interleukin 2 receptors as a differentiation marker on intrathymic stem cells. *Nature* **314**, 98-100.

Ceredig R., and Waltzinger C. (1990). Neonatal mouse CD4+ mature thymocytes show responsiveness to interleukin 2 and interleukin 7: Growth in vitro of negatively selected V$\beta$6- and V$\beta$11-expressing CD4+ cells from (C57BL/6 × DBA/2)F1 mice. *Int. Immunol.* **2**, 869-877.

Crook K., and Hunt S.V. (1996). Enrichment of early fetal-liver hemopoietic stem cells of the rat using monoclonal antibodies against the transferrin receptor, Thy-1 and MRC-OX82. *Dev. Immunol.* **4**, 235-246.

Deans J.P., Wilkins J.A., Caixa S., Pruski E., and Pilarski L.M. (1991). Prolonged expression of high molecular mass CD45RA isoform during the differentiation of human progenitor thymocytes to CD3+ cells in vitro. *J. Immunol.* **147**, 4060-4068.

Fedecka-Brunner B., Penninger J., Vaigot P., Lehman A., Martinez A. C., and Kroemer F. (1991). Development expression of IL-2R light chain (CD25) in the chicken embryo. *Dev. Immunol.* **1**, 237-242.

Fowlkes B.J., Kruisbeek A., Ton-That H., Weston M.A., Coligan J.E., Schwartz R.H., and Pardoll D.M. (1987). A novel population of T-cell receptor $\alpha\beta$-bearing which predominantly expresses a single V$\beta$ gene family. *Nature* **329**, 251-253.

Godfrey D.I., Kennedy J., Suda T., and Zlotnik A. (1993). A development pathway involving four phenotypically and functional distinct subsets of CD3-4-8-triple-negative adult mouse thymocytes defined by CD44 and CD25 expression. *J. Immunol.* **150**, 4244-4252.

Goff L.K., Larsson L., and Fisher A.G. (1990). Expression of high molecular weight isoforms of CD45 by mouse thymic progenitor cells. Eur. *J. Immunol.* **20**, 665-671.

Gotlieb W.H., Bristol L.A.,'Weissman A.M., Durum S.K., Tackacs L. (1993). Up-regulation of T cell receptor $\gamma$-chain transcription by interleukin-2. *Cell. Immunol.* **151**, 345-350.

Groettnrup M., Undewiss K., Azogni R., Owen M.J., Harday A.C., and von Boehmer H. (1993). A novel disulfide-linked heterodimer on pro-T cells consist of the T-cell receptor $\beta$ chain and a 33KD glycoprotien. *Cell* **75**, 283-294.

Habu S., Okumura K., Diamantein T., and Shevach E.M. (1985). Expression of IL-2R on murine fetal thymocytes. *Eur. J. Immunol.* **15**, 456-460.

Haynes B.F., and Heinly C.S. (1995). Early human T cell development: Analysis of the human thymus at the time of initial entry of hematopoietic stem cells into the fetal thymic microenvironment. *J. Exp. Med.* **181**, 1445-1458.

He W., and Kabelitz D. (1993). Differential effects of interleukin-7 and interleukin-2 on T-cell receptor $\gamma\delta$-expressing cells within CD4−CD8− postnatal human thymocytes. *Int. Arch. Allergy Immunol.* **102**, 321-326.

Hosseinzadeh H., and Goldschneider Y. (1993). Recent thymic emigrants in the rat express a unique antigenic phenotype and undergo post-thymic maturation in peripheral lymphoid tissues *J. Immunol.* **150**, 1670-1679.

Hünig T., Tiefenthaler G., Schlipköter E., and Lawetzky A. (1989a). The T-cell antigen receptor and CD2 in rat T-cell activation and ontogeny. In Progress in Immunology, Melchers F., Ed. (Berlín: Springer-Verlag), pp. 139-146.

Hünig T., Wallny H.-J., Hartley J.K., Lawetzky A., and Tiefenthaler G. (1989b). A monoclonal antibody to a constant determinant of the rat T cell antigen receptor that induces T cell activation. *J. Exp. Med.* **169**, 73-86.

Jotereau F., Henze F., Salomon-Vie V., and Gascan H. (1987). Cell kinetics in the fetal mouse thymus: Precursor cell input, proliferation and emigration. *J. Immunol.* **138**, 1026-1030.

Kampinga J., and Aspinall R. (1990). Thymocyte differentiation and thymic microenvironment development in the fetal rat thymus: An immunohistological approach. In Thymus Uptade, Kendall, M. D., and Ritter, M., Eds. (London: Harwood Academic Publishers), pp. 149-186.

Kondo M., Takeshita T., Ishii N., Nakamura M., Watanabe S., Arai K., and Sagamura K. (1993). Sharing of the interleukin-2 (IL-2) receptor $\gamma$ chain between receptors for IL-2 and IL-4. *Science* **262**, 1874-1877.

Law D.A., Spruyt L.L., Paterson D.J., and Williams A.F. (1989). Subsets of thymopoietic rat thymocytes defined by expression of the CD2 antigen and MRC OX22 determinant of the leukocyte-common antigen CD45. *Eur. J. Immunol.* **19**, 2289-2295.

Lawetzky A., Kubbies M., and Hünig T. (1991). Rat first-wave mature thymocytes: Cycling lymphoblast that are sensible to activation-induced cell death but rescued by IL-2. *Eur. J. Immunol.* **21**, 2599-2604.

Levelt C.N., and Eichmann K. (1995). Receptors and signals in early thymic selection. *Immunity* **3**, 667-672.

Mertsching E., and Ceredig R. (1996). T cell receptor-$\gamma\delta$-expressing fetal mouse thymocytes are generated without T cell receptor V$\beta$ selection. *Eur. J. Immunol.* **26**, 804-810.

Modigliani Y., Coutinho G., Burlen-Defranoux O., Coutinho A., and Bandeira A. (1994). Differential contribution of thymic outputs and peripheral expansion in the development of peripheral T cell pool. *Eur. J. Immunol.* **24**, 1223-1227.

Park I.-H., and Hünig T. (1995). Regulation of RAG-1, TcR, and IL-2RmRNA expression in a cohort of synchronously differentiating rat CD4+CD8+ thymocytes. The 9th International Congress of Immunology, Abstract A-2945. San Francisco, July 23-25, 1995. San Francisco: American Association of Immunologists p. 497.

Paterson D.J., and Williams A.F. (1987). An intermediate cell in thymocyte differentiation that expresses CD8 but not CD4 antigen. *J. Exp. Med.* **166**, 1603-1608.

Penit C., and Vasseur F. (1989). Cell proliferation and differentiation in the fetal and early postnatal mouse thymus. *J. Immunol.* **142**, 3369-3377.

Ritter M.A., Gordon L.K., and Goldschneider I. (1978). Distribution and identification of Thy 1 bearing cells ontogeny in rat hemopoietic and lymphoid tissues. *J. Immunol.* **121**, 2463-2471.

Shortman K., and Wu L. (1996). Early T lymphocyte progenitors. *Annu. Rev. Immunol.* **14**, 29-47.

Takacs L., Ruscetti F.W., Kovacs E.J., Rocha B., Brocke S., Diamantstein T., and Mathieson B.J. (1988). Immature double negative (CD4−CD8−) rat thymocytes do not express IL-2 receptors. *J. Immunol.* **141**, 3810-3818.

Toribio M.L., Gutierrez J.C., Pezzi L., Marcos M.A., and Martinez A.C. (1989). Interleukin-2-dependent autocrine proliferation in T-cell development. *Nature* **342**, 82-85.

Varas A., Vicente A., Jiménez E., Alonso L., Moreno J., Muñóz J.J., Zapata A.G. (1997a). Interleukin-7 treatment promotes the differentiation pathway of T-cell-receptor-$\alpha\beta$ cells selectively to the CD8+ cell lineage. *Immunology* **92**, 457-464.

Varas A., Vicente A., Romo T., and Zapata A.G. (1997b). Role of IL-2 in rat fetal thymocyte development. *Int. Immunol.* **9**, 1589-1599.

Vicente A., Varas A., Alonso L., Gómez del Moral M., and Zapata A.G. (1994). Ontogeny of rat thymic dendritic cells. *Immunology* **82**, 75-81.

Vicente A., Varas A., Moreno J., Sacedón R., Jiménez E., and Zapata A.G. (1995). Ontogeny of rat thymic macrophages: Phenotypic characterization and possible relationships between different cell subsets. *Immunology* **85**, 99-105.

Vicente A., Varas A., Sacedón R., and Zapata A.G. (1996). Histogenesis of the epithelial component of rat thymus: An ultrastructural and immunohistological analysis. *The Anatom. Rec.* **244**, 506-519.

Weissman I.L. (1967). Thymus cell migration. *J. Exp. Med.* **126**, 291-294.

Wilson A., and MacDonald H.R. (1995). Expression of genes encoding the pre-TcR and CD3 complex during thymus development. *Int. Immunol.* **7**, 1659-1664.

Zuñiga-Pflücker J.C., and Lenardo M.J. (1996). Regulation of thymocyte development from immature progenitors. *Curr. Opin. Immunol.* **8**, 215-224.

